# Novel protein-based prognostic signature linked to immunotherapeutic efficiency in ovarian cancer

**DOI:** 10.1186/s13048-024-01518-w

**Published:** 2024-09-28

**Authors:** Shuo-Fu Chen, Liang-Yun Wang, Yi-Sian Lin, Cho-Yi Chen

**Affiliations:** 1 Department of Heavy Particles & Radiation Oncology, Taipei Veterans General Hospital, Taipei, 112 Taiwan; 2 Institute of Biomedical Informatics, National Yang Ming Chiao Tung University, Taipei, 112 Taiwan; 3 Program in Genetics and Genomics, Baylor College of Medicine, Houston, TX 77030 USA; 4 Brain Research Center, National Yang Ming Chiao Tung University, Taipei, 112 Taiwan

**Keywords:** Ovarian cancer, Immunotherapy, Proteomics, Prognosis, Tumor immune microenvironment

## Abstract

**Background:**

Personalized medicine remains an unmet need in ovarian cancer due to its heterogeneous nature and complex immune microenvironments, which has gained increasing attention in the era of immunotherapy. A key obstacle is the lack of reliable biomarkers to identify patients who would benefit significantly from the therapy. While conventional clinicopathological factors have exhibited limited efficacy as prognostic indicators in ovarian cancer, multi-omics profiling presents a promising avenue for comprehending the interplay between the tumor and immune components. Here we aimed to leverage the individual proteomic and transcriptomic profiles of ovarian cancer patients to develop an effective protein-based signature capable of prognostication and distinguishing responses to immunotherapy.

**Methods:**

The workflow was demonstrated based on the Reverse Phase Protein Array (RPPA) and RNA-sequencing profiles of ovarian cancer patients from The Cancer Genome Atlas (TCGA). The algorithm began by clustering patients using immune-related gene sets, which allowed us to identify immune-related proteins of interest. Next, a multi-stage process involving LASSO and Cox regression was employed to distill a prognostic signature encompassing five immune-related proteins. Based on the signature, we subsequently calculated the risk score for each patient and evaluated its prognostic performance by comparing this model with conventional clinicopathological characteristics.

**Results:**

We developed and validated a protein-based prognostic signature in a cohort of 377 ovarian cancer patients. The risk signature outperformed conventional clinicopathological factors, such as age, grade, stage, microsatellite instability (MSI), and homologous recombination deficiency (HRD) status, in terms of prognoses. Patients in the high-risk group had significantly unfavorable overall survival (*p* < 0.001). Moreover, our signature effectively stratified patients into subgroups with distinct immune landscapes. The high-risk group exhibited higher levels of CD8 T-cell infiltration and a potentially greater proportion of immunotherapy responders. The co-activation of the TGF-β pathway and cancer-associated fibroblasts could impair the ability of cytotoxic T cells to eliminate cancer cells, leading to poor outcomes in the high-risk group.

**Conclusions:**

The protein-based signature not only aids in evaluating the prognosis but also provides valuable insights into the tumor immune microenvironments in ovarian cancer. Together our findings highlight the importance of a thorough understanding of the immunosuppressive tumor microenvironment in ovarian cancer to guide the development of more effective immunotherapies.

**Supplementary Information:**

The online version contains supplementary material available at 10.1186/s13048-024-01518-w.

## Introduction

Ovarian cancer (OC) is the leading cause of death among gynecological malignancies [1, 2]. More than 207,000 women worldwide die from OC each year. It is estimated that by 2040, the annual new cases of OC would rise by 42% to 445,721, and the number of deaths from OC each year is expected to increase to 313,617, a rise of over 50% from 2020 [3, 4]. The high-grade serous ovarian cancer, which accounts for 70% of epithelial ovarian cancers, is the most common and lethal subtype [1]. It is challenging that many high-grade serous OC patients remain undiagnosed until advanced stages due to their non-specific symptoms. Currently, the mainstay of OC treatment consists of optimal debulking surgery followed by platinum-based chemotherapy [5]. However, up to 70% of OC patients eventually experience recurrence [5]. Compared to other types of cancers, the mortality rates for OC have shown minimal improvement in recent decades [2]. This necessitates the exploration of alternative therapeutic options, including immunotherapy.

While immunotherapy has shown promise in treating various cancers, its efficacy in OC is still modest, with an overall response rate of 15% with nivolumab [6, 7]. The limited efficacy of immunotherapy in OC may be attributed to the diverse nature of the disease. There is an urgent need for reliable biomarkers to classify patients based on their treatment responsiveness and facilitate personalized medicine. While clinicopathological characteristics, such as staging and histological grades, are important prognostic indicators in OC, they provide limited information for assessing immunotherapy responses [8]. Some of the genetic aberrations have been recognized as potential biomarkers for immunotherapy response in other cancer types, including microsatellite instability (MSI), tumor mutation burden (TMB), and homologous recombination deficiency (HRD). Despite the success in other cancers, these biomarkers were shown not as effective in OC [8]. For example, among non-immune checkpoint inhibitor (ICI)-treated patients, a higher TMB did not correlate with an improved prognosis and, in fact, was associated with worse survival in several cancer types [9]. Moreover, no significant differences in survival were observed among OC patients based on MSI status, TMB levels, or neoantigen loads [9, 10]. HRD, despite being associated with the clinical prognosis of OC patients, exhibited only a weak positive correlation with T cell-inflamed activity (TCIA) and no correlation with the expression levels of CD8 or PD-L1 [11, 12]. Also, there is insufficient evidence that OC patients with higher HRD levels would benefit from pembrolizumab [13, 14].

In addition to conventional biomarkers, recent advancements in high-throughput technology have opened up new possibilities for improved risk assessment and personalized treatment strategies for OC patients [15–17]. Among these technologies, proteomics offers advantages over genomics and transcriptomics as it directly identifies functional effector molecules involved in the pathophysiology of OC, and the variations in gene expression levels do not always translate to functional differences [18, 19]. Moreover, several proteomic models have shown promise in distinguishing between immune-hot and immune-cold tumors [20]. Increasing studies have emphasized the significant role of proteins in shaping the compositions and activation states of the immune landscapes [20, 21]. One promising technique, Reverse Phase Protein Array (RPPA), enables sensitive and cost-effective functional proteomic profiling of samples from TCGA [22, 23]. A comprehensive study on protein expression is crucial for investigating the interplay between the immune system and tumor cells, providing valuable insights for clinical outcomes and guiding treatment decisions [24]. Therefore, establishing a protein-based prognostic signature for immunotherapy responses would be of great clinical significance.

In this study, we developed a protein-based signature for evaluating the prognoses and immunotherapy responses in OC patients. We validated the performance of our risk score in the training and test sets. Our finding demonstrated that the five-protein prognostic signature effectively divided OC patients into subgroups with distinct immune landscapes and prognoses based on RPPA profiles. Furthermore, results obtained with multiple established signatures for immunotherapy supported the roles of these proteins in determining the immunomodulatory features of OC tumors. Our study highlights the potential of the protein-based signature as a promising new approach for predicting prognoses in OC patients, providing novel insights into the tumor immune microenvironments and treatment decisions.

## Methods

### Dataset acquisition and pre-processing

The RPPA protein expression profile (log2 transformed values) and RNA-sequencing data (FPKM values) of OC patients were obtained from The Cancer Proteome Atlas (TCPA) database (https://tcpaportal.org/tcpa/*)* [22] and the pan-cancer atlas of The Cancer Genome Atlas (TCGA) program (https://gdc.cancer.gov/about-data/publications/pancanatlas*)* [25]. The tumor specimens were retrieved from cytoreductive surgery before the initiation of chemotherapy. The corresponding clinical information and mutation profile, including TMB, MSI, and HRD, were retrieved from the TCGA pan-cancer immune landscape (https://gdc.cancer.gov/about-data/publications/panimmune*)* [26]. The TMB, MSI, and HRD were calculated using formulas as previously described [26].

### Screening of immune-related proteins in OC

To identify immune-related RPPA proteins, we first performed a clustering analysis on OC patients using the single-sample gene set enrichment analysis (ssGSEA) scores of 29 immune-related gene sets (Supplementary Table S1) [27, 28]. The patients were clustered into two immune subtypes using hierarchical clustering with Euclidean distance and Ward’s linkage. The ssGSEA analysis was conducted using the “GSVA” R package [29]. The RPPA proteins associated with immune subtypes were selected through elastic net regularization with an alpha value of 0.1 followed by a 10-fold cross-validation in the “glmnet” R package [30, 31]. The RPPA proteins with non-zero coefficients were considered immune-related proteins for subsequent analysis.

### Construction of the protein prognostic signature

A total of 374 OC patients with complete RPPA data and survival information were included in this study. The patients were randomly divided into training (*n* = 202) and test (*n* = 172) cohorts using the “Splitstackshape” package [32], with stratification based on age, stage, and grade at a ratio of 5:5. The baseline information of our OC cohort was summarized in Table 1. There was no significant difference in clinical and molecular characteristics between the training and the test groups. The training group was utilized to explore potential prognostic proteins for OC patients and construct the prognostic risk score, which was further validated by the test group. The immune-related RPPA proteins from the previous screening were subjected to univariate Cox regression, and proteins with a p-value < 0.05 were considered statistically significant for subsequent LASSO regression analysis. The optimal penalty parameter (λ) for LASSO regression analysis was determined by the 10-fold cross-validation using the “glmnet” R package [30, 31]. Finally, proteins with nonzero regression coefficients were eligible for multivariate Cox regression analysis, and those with independent prognostic values were enrolled to construct a risk score by summing their expression levels multiplied by their respective coefficients. The patients were stratified into high- and low‐risk groups based on the median value of the risk scores. Patients with a risk score higher than the median value were considered the high-risk group. The protein expression profiles and the risk group for each patient were visualized using the “ComplexHeatmap” R package [33].

**Table 1:** Clinical and molecular characteristics of the study population

	Entire set *n* = 374	Training set *n* = 202	Test set *n* = 172	*p* value
Age (years), median (IQR)	59 (52–69)	59 (52–69)	58.5 (51–68)	0.57
Stage				0.92
I, n (%)	15 (4)	8 (4)	7 (4)	
II, n (%)	26 (7)	14 (7)	12 (7)	
III, n (%)	287 (77)	155 (77)	135 (78)	
IV, n (%)	42 (11)	22 (11)	20 (12)	
Unknown, n (%)	4 (1)	3 (2)	1 (1)	
Grade				0.25
II, n (%)	43 (11)	20 (10)	23 (13)	
III, n (%)	326 (87)	180 (89)	146 (85)	
Unknown, n (%)	5 (1)	2 (1)	3 (2)	
TMB, median (IQR)	2.07 (0.94–2.92)	2.00 (1.12–2.86)	1.97 (0.61–2.93)	0.57
MSI, median (IQR)	0.84 (0.42–1.41)	0.97 (0.51–1.50)	0.79 (0.32–1.29)	0.05
Aneuploidy, median (IQR)	13 (7–20)	13 (7–20)	14 (7.5–20)	0.63
HRD, median (IQR)	46 (30–64)	46 (32–63)	44.5 (29.3–64)	0.63

### Performance assessment of the risk score

The Kaplan-Meier (K-M) survival curves and the log-rank test were performed to compare the survival outcomes between high- and low-risk groups. The performance of the prognostic signatures was assessed by the area under the time-dependent receiver operating characteristic (ROC) curve. The area under the ROC curve (AUC) at 1, 3, and 5 years was plotted using the “survivalROC” R package [34]. To assess the independent prognostic value of the risk score from other clinical variables, multivariable Cox regression analyses were performed in the training and test groups.

### Clinical relevance and enrichment analysis of the associated proteins

The correlations between the risk score, five prognostic proteins, and clinicopathological variables were statistically evaluated using Spearman’s correlation coefficients. The biological network of the five proteins and their functionally similar genes was constructed by GeneMANIA (http://genemania.org/*)* [35]. To further investigate the biological significance of the risk score, co-expressed proteins associated with the five proteins in our risk model were identified using Pearson correlation analysis, with the criteria of a “rho > 0.3” and a significance level of “p < 0.001”. The genes responsible for these co-expressed proteins were subjected to functional and pathway enrichment analysis using the “clusterProfiler” R package [36]. The pathway and functional annotation of the risk score-associated genes was performed through the Kyoto Encyclopedia of Genes and Genomes (KEGG) [37] and Gene Ontology (GO) databases, including cellular component (CC), biological process (BP), and molecular function (MF) [38].

### Deciphering the immune landscape between risk groups

To characterize the tumor immune microenvironments, a panel of 77 gene expression signatures representing immune checkpoints, antigen presentation, lymphocyte infiltration, interferon pathway, wound response, and ECM (extracellular matrix) dysregulation were scored for each sample. These scores were obtained from the TCGA immune landscape (https://gdc.cancer.gov/about-data/publications/panimmune*)* to thoroughly compare the gene expression and immune cell abundance between the risk groups [26]. Additionally, the CIBERSORT algorithm was applied to elucidate the differences in the immune cell populations between the risk groups [39]. The generated results with *p* < 0.05 were eligible for subsequent analysis. The correlations between the risk score and infiltrating immune cells were statistically evaluated using Spearman’s correlation.

### Assessment of immunotherapy responses

Using various established signatures for immunotherapy responses, we were able to classify the OC patients as responders or non-responders and calculated the odds ratio (OR) to assess the discriminative performance of the risk score. The Tumor Immune Response Signature Finder (TIRSF) web application (http://tirsf.renlab.org/*)* offers a comprehensive collection of transcriptomic signatures associated with immunotherapy responses [40]. Most of these signatures have been validated in multiple cancer types. For each signature, the gene expression counts were input, and the scores were computed according to the description in original publications (Supplementary Table S2).

Next, Immune Cell Abundance Identifier (ImmuCellAI) (http://bioinfo.life.hust.edu.cn/ImmuCellAI/*)* was used to estimate the ability of the risk score to evaluate the responses to immunotherapy [41]. ImmuCellAI is an SVM (support vector machine)-based computational model that uses gene expression data to estimate the abundance of 24 different immune cells and immunotherapy responses. Finally, the Tumor Immune Dysfunction and Exclusion (TIDE) (http://tide.dfci.harvard.edu/*)* algorithm was employed to establish a tumor immune evasion model and predict immunotherapy responses by integrating potential regulators restricting T cell function or infiltration [42].

### Statistical analysis

All the statistical and computational analyses were conducted using the R statistical software environment (version 4.2.1). Wilcoxon rank-sum test was used to derive the p-value for continuous variables, and Fisher’s exact test was applied for categorical features. A p-value < 0.05 was considered statistically significant.

## Results

### Construction of a proteomic prognostic signature

**Fig. 1:**
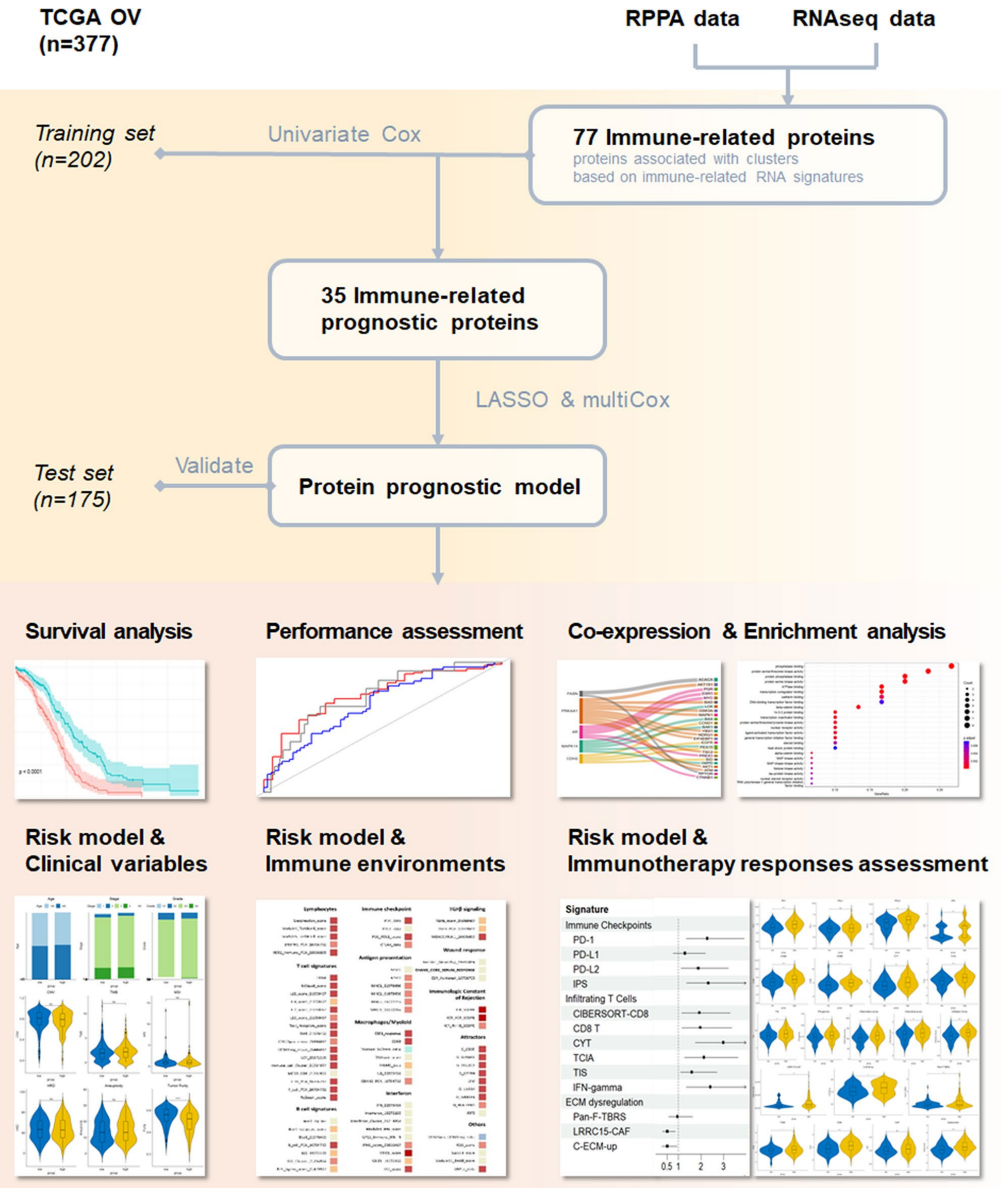
The workflow of this study

**Fig. 2:**
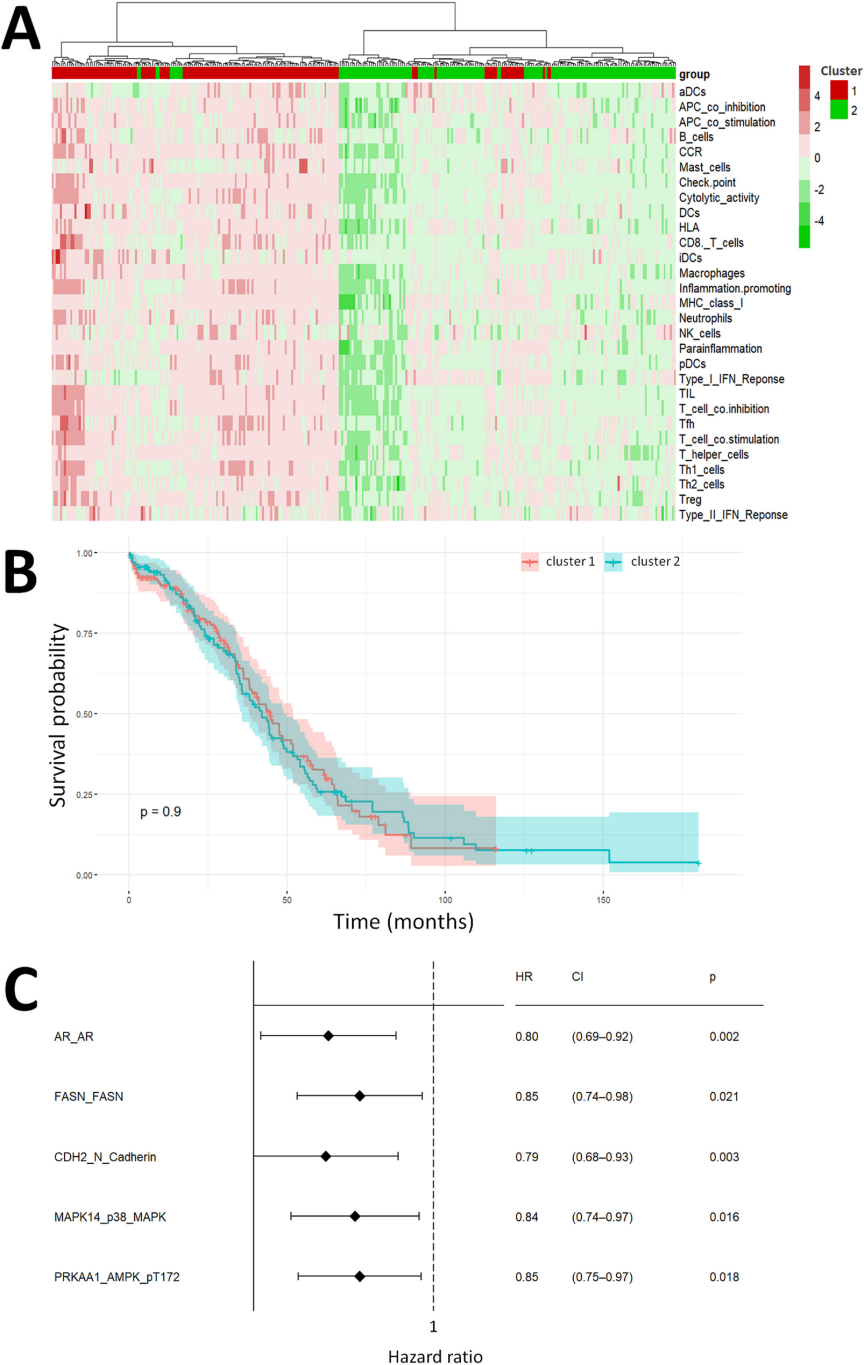
Identification of immune-related proteins from RNA-based signatures. (**A**) Hierarchical clustering based on the ssGSEA values of transcriptomic immune signatures. (**B**) The Kaplan–Meier analysis of overall survival between the two immune clusters defined by transcriptomic signatures. (**C**) Forest plot showing the hazard ratio of each protein in the multivariable Cox regression analysis

Our study aims to develop a protein-based signature for assessing prognoses and immunotherapy responses in OC. The workflow of our study is illustrated in Fig. 1. First, we conducted a hierarchical clustering analysis on OC patients using the ssGSEA scores of 29 RNA-based immune signatures. The resulting clusters were characterized by distinct distributions of the representative signatures (Fig. 2A). However, there were no significant differences in overall survival between the two immune clusters defined by transcriptomic immune signatures (Fig. 2B). Therefore, we proceeded to explore the potential of protein-based signatures in predicting prognostic outcomes. We utilized an elastic net regression model as our initial screening, and 77 RPPA proteins that showed robust associations with the immune clusters were identified. Next, these immune-related RPPA proteins were subjected to univariate Cox analysis to evaluate their prognostic significance. Out of them, 35 were found to be significantly associated with survival in the training set (*p* < 0.05) and were chosen as candidate proteins for signature construction. Finally, by applying the LASSO penalized Cox regression model and multivariate Cox regression analyses to these proteins in the training set, we constructed a prognostic signature consisting of 5 immune-related RPPA proteins, including AR, FASN, CDH2, MAPK14, and PRKAA1. Among these proteins, AR had the largest negative coefficients, suggesting a greater potential for discrimination. The risk score for each patient was then calculated using the Cox coefficients for each RPPA protein, as follows:

$$\eqalign{ Risk score = & - 0.231888021 *AR \cr & - 0.143034677 *FASN \cr & - 0.163957462 *CDH2 \cr & - 0.132508744 * MAPK14 \cr & - 0.176161691 * PRKAA1 \cr}$$

The forest plot of hazard ratio suggests that all five proteins (AR, FASN, CDH2, MAPK14, and PRKAA1) were favorable prognostic proteins (Fig. 2C).

### Performance assessment of the prognostic signature

**Fig. 3:**
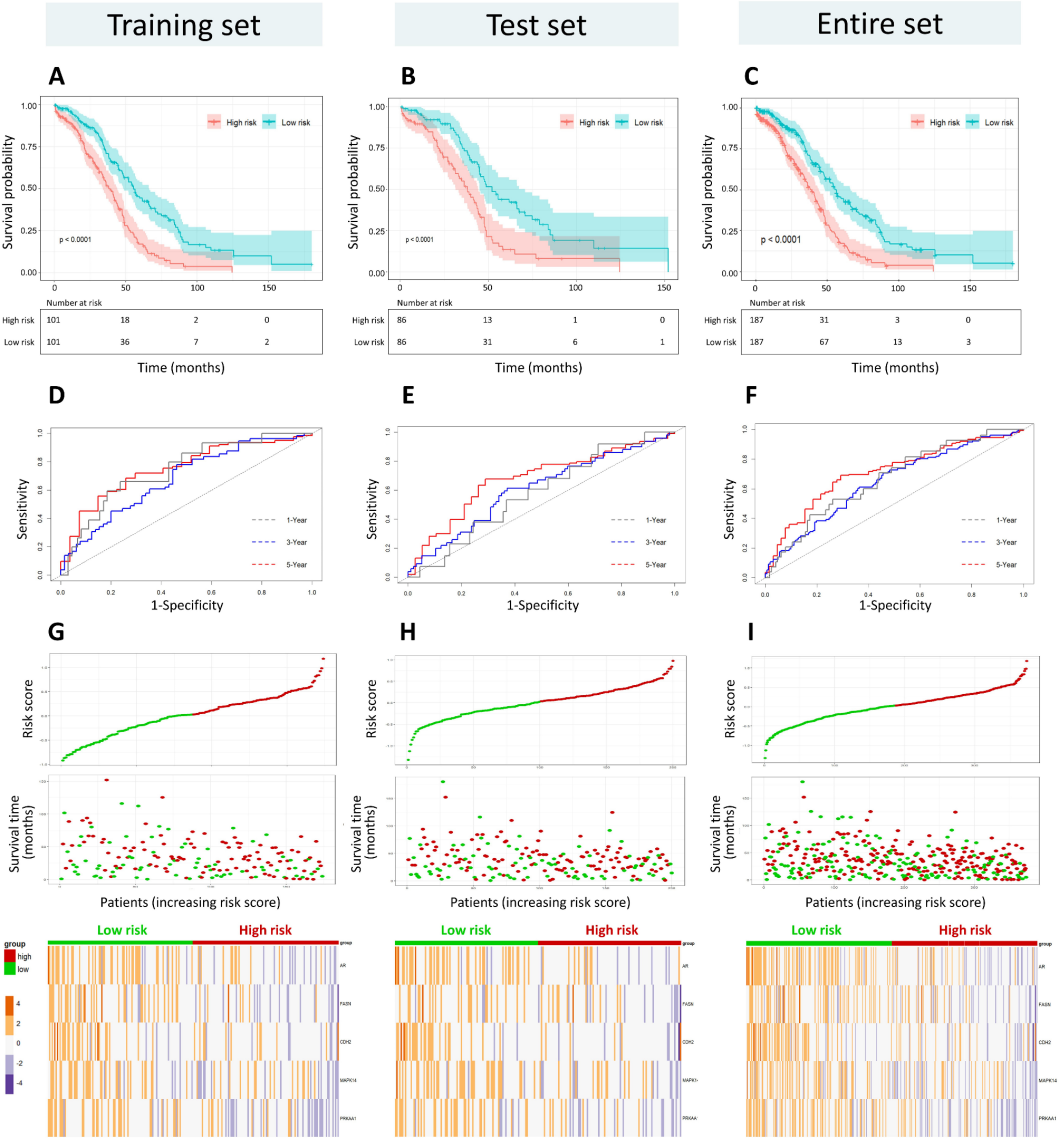
Performance assessment of the prognostic signature in the training set (left), the test set (middle), and the entire set (right). (**A**-**C**) The Kaplan–Meier analysis of overall survival in the high-risk and low-risk groups. (**D**-**F**) The time-dependent ROC analysis of risk scores for predicting overall survival. (**G**-**I**) The distribution of risk scores, survival status, and five-protein expression profiles for OC patients. The columns of heatmaps were ordered by increasing risk scores

Patients in the training and test cohorts were divided into high-risk and low-risk groups based on the median risk score. Apart from the risk scores, there were no significant differences in other clinical characteristics between the high-risk and low-risk groups in both the training and test datasets (Supplementary Table S3). The Kaplan–Meier survival analysis was conducted to evaluate the impact of the risk score on the prognosis. In both the training and test cohorts, patients in the high-risk group had significantly shorter overall survival than those in the low-risk group, indicating that the risk scores are adversely correlated to the prognostic outcomes of OC patients (Fig. 3A C). The test cohort and the entire cohort were used to validate the prognostic power of the risk score. The ROC analysis showed that the 1-, 3- and 5-year AUC were 0.74, 0.68, and 0.75 in the training cohort and 0.58, 0.61, and 0.68 in the test cohort, respectively (Fig. 3D and E). Across the entire dataset, the high-risk group consistently exhibited worse prognostic outcomes at different disease stages, and the 1-, 3-, and 5-year AUC were 0.67, 0.65, and 0.71, respectively (Fig. 3F). The distribution of risk score, survival status, and protein expression profiles remained consistent across the training, test, and entire cohort (Fig. 3G and I). In both training and test cohorts, expressions of the five proteins (AR, FASN, CDH2, MAPK14, and PRKAA1) were significantly downregulated in the high-risk group.

### The risk score is an independent prognostic factor in OC patients

**Fig. 4:**
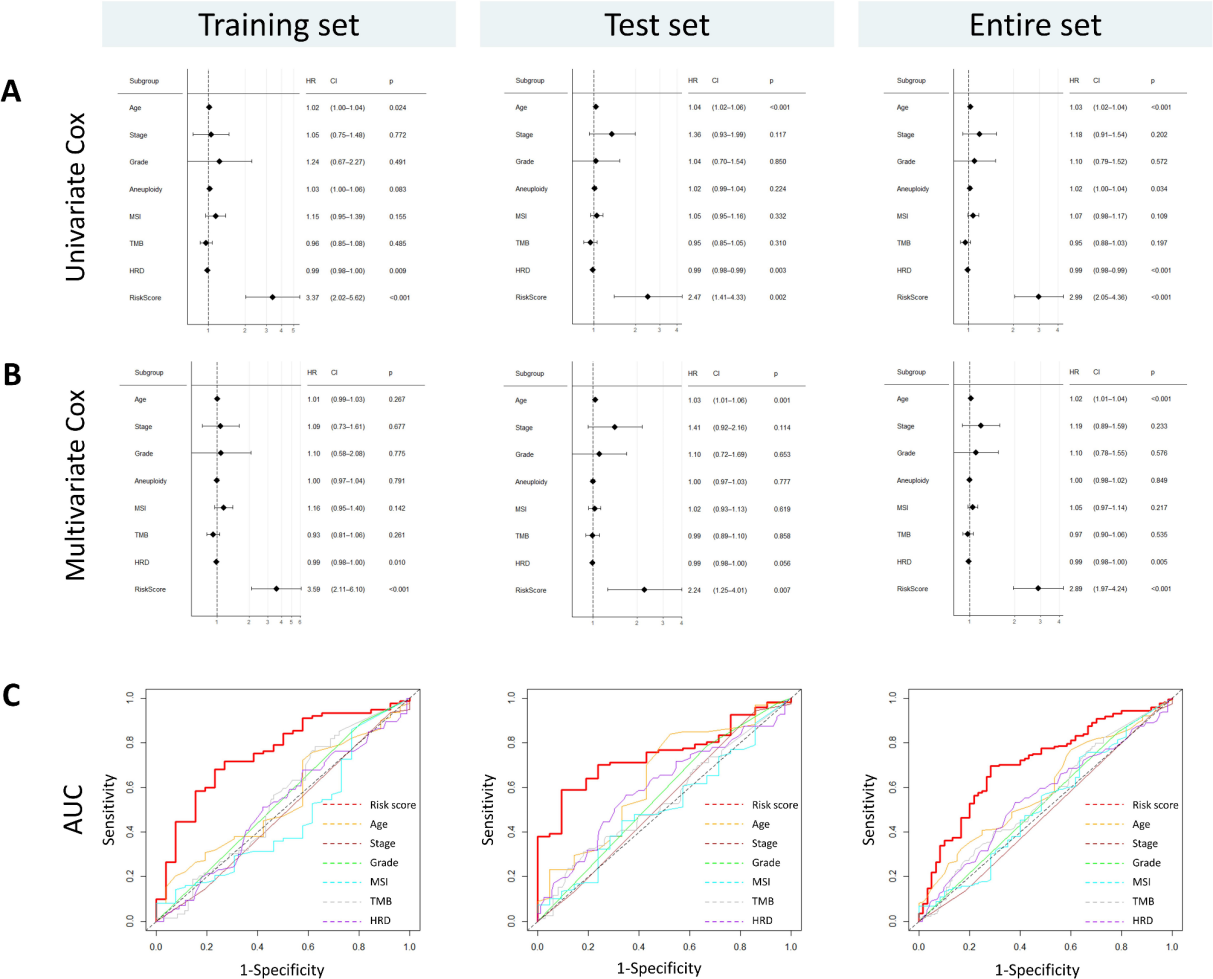
The proteomic signature has better prognostic performance than other clinicopathological factors, as shown in (**A**) univariate Cox regression, (**B**) multivariate Cox regression, and (**C**) ROC analysis

To determine the independent prognostic value of the risk score compared with other clinicopathological parameters, univariate and multivariate Cox regression analyses were performed in the training and test sets. The univariate Cox regression demonstrated that the risk score (HR = 2.99, *p* < 0.001), age (HR = 1.03, *p* < 0.001), and HRD (HR = 0.99, *p* < 0.001) were significantly associated with the prognosis of OC patients (Fig. 4A). The risk score remained as an independent prognostic factor in the training and test dataset after adjusting for other clinical and molecular characteristics, including age, grade, stage, TMB, MSI, and HRD (HR = 2.89, *p* < 0.001) (Fig. 4B). The AUC of our protein signature outperformed existing clinicopathological characteristics such as age, grade, stage, TMB, MSI, and HRD (Fig. 4C). It is worth noting that while age was not an independent factor in the training set during multivariate analysis, it remained independent in both the test and entire datasets. Combining age with the protein signature resulted in a more robust predictive model (Supplementary Figure S1).

### Association between the risk score and clinical variables

**Fig. 5:**
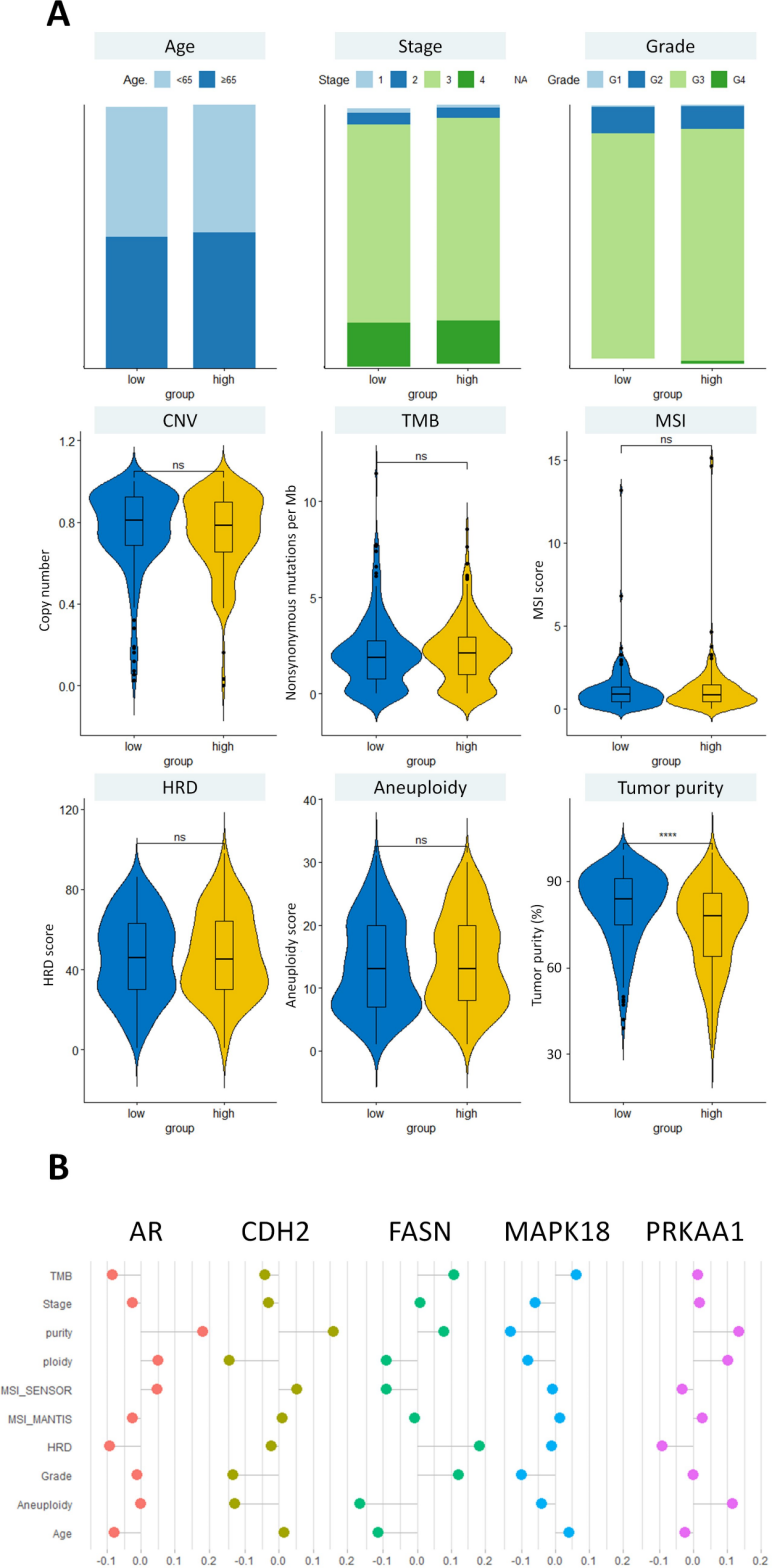
The association between the risk score, the five prognostic proteins, and clinico-genomic characteristics. (**A**) Stacked bar plots depicting the proportions of clinical variables (age, stage, grade) in the high- and low-risk groups. Violin plots comparing the distribution of genomic features between the risk groups using the Wilcoxon rank sum test. **** p < 0.0001, *ns = non-significant* (**B**) Lollipop plot illustrating the Spearman’s correlation coefficients between the protein expression levels and clinical features

Next, we explored the association between the risk score and the clinicopathological variables in OC patients. The two risk groups did not differ significantly in terms of clinical stage, pathological grade, TMB, MSI, or HRD (Fig. 5A). However, we observed a distinct pattern in tumor purity, where the high-risk group exhibited lower tumor purity compared to the low-risk group. This implied a higher proportion of non-cancerous cells, including immune cells and fibroblasts, in the tumor microenvironments of the high-risk group. Among the five proteins in the risk score, AR, CDH2, and PRKAA1 were more highly expressed in samples with higher tumor purity (Fig. 5B). The expression of PRKAA1 exhibited a positive association with aneuploidy, while FASN and CDH2 displayed a negative association. Tumors with higher grades and HRD showed increased expression levels of FASN. Conversely, tumors with higher grades and aneuploidy demonstrated decreased expression of CDH2.

### Potential biological pathways associated with the risk score

**Fig. 6:**
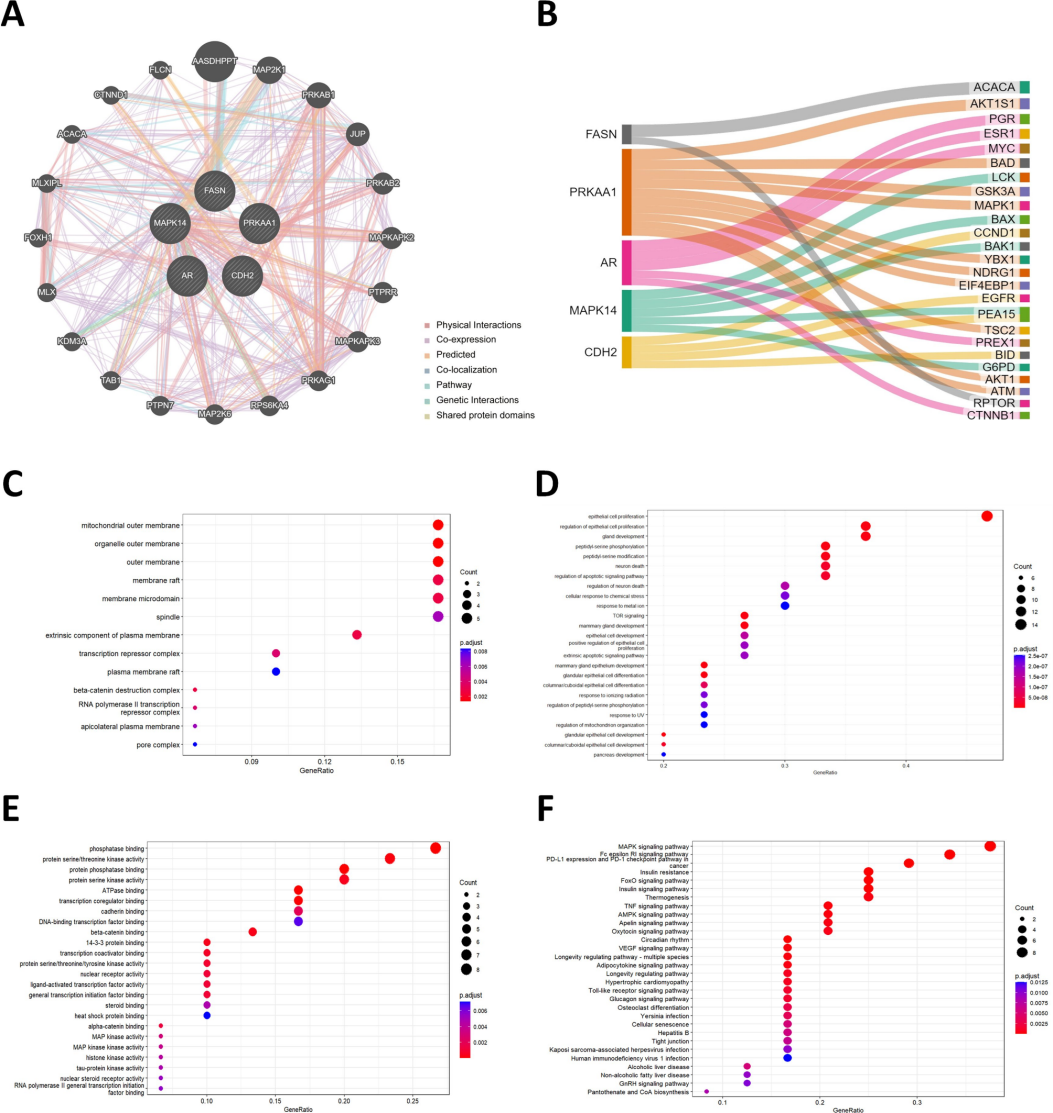
Co-expression and enrichment analysis based on the risk score. (**A**) The protein-protein interaction networks, including the physical interaction, co-expression, predicted, co-localization, common pathway, genetic interaction, and shared protein domains, were constructed by GeneMANIA. (**B**) Sankey diagram summarizing the co-expressed proteins with the five prognostic proteins in the risk score. These co-expressed proteins were subjected to enrichment analysis based on (**C**) Gene Ontology Cellular Component (GO CC), (**D**) Gene Ontology Biological Process (GO BP), (**E**) Gene Ontology Molecular Function (GO MF), and (**F**) KEGG database

The biological network of the five prognostic proteins and their functionally relevant genes is displayed in Fig. 6A. To gain a deeper understanding, we identified co-expressed proteins that showed significant correlations with these five proteins. Specifically, FASN had a significant positive correlation with ACACA (*r* = 0.518, *p* < 0.001). AR was positively correlated with PGR (*r* = 0.425, *P* < 0.001) and ESR1 (*r* = 0.416, *P* < 0.001). PRKAA1 had a significant positive correlation with AKT1S1 (*r* = 0.461, *P* < 0.001) and BAD (*r* = 0.405, *P* < 0.001). The expression of MAPK14 was significantly related to LCK (*r* = 0.401, *p* < 0.001). As for CDH2, CCND1 and EGFR were the top and second significantly co-expressed proteins, respectively. The Sankey plot visually summarizes the co-expression network between the proteins (Fig. 6B).

To explore the biological functions of the co-expressed proteins, we performed enrichment analysis using their corresponding genes. The GO analysis revealed that these co-expressed proteins were predominantly membrane proteins (CC), involved in functions related to phosphatase binding and serine/threonine kinase activity (MF), and participated in various biological processes such as epithelial cell proliferation and regulation of apoptosis (BP). Furthermore, KEGG analysis supported their involvement in several immune-related pathways, including the MAPK signaling pathway, Fc epsilon RI-mediated signaling pathway, and PD-L1 expression/PD-1 checkpoint pathway in cancer (Fig. 6C-F).

### The risk score is associated with CD8 T immunity and inflamed phenotypes

**Fig. 7:**
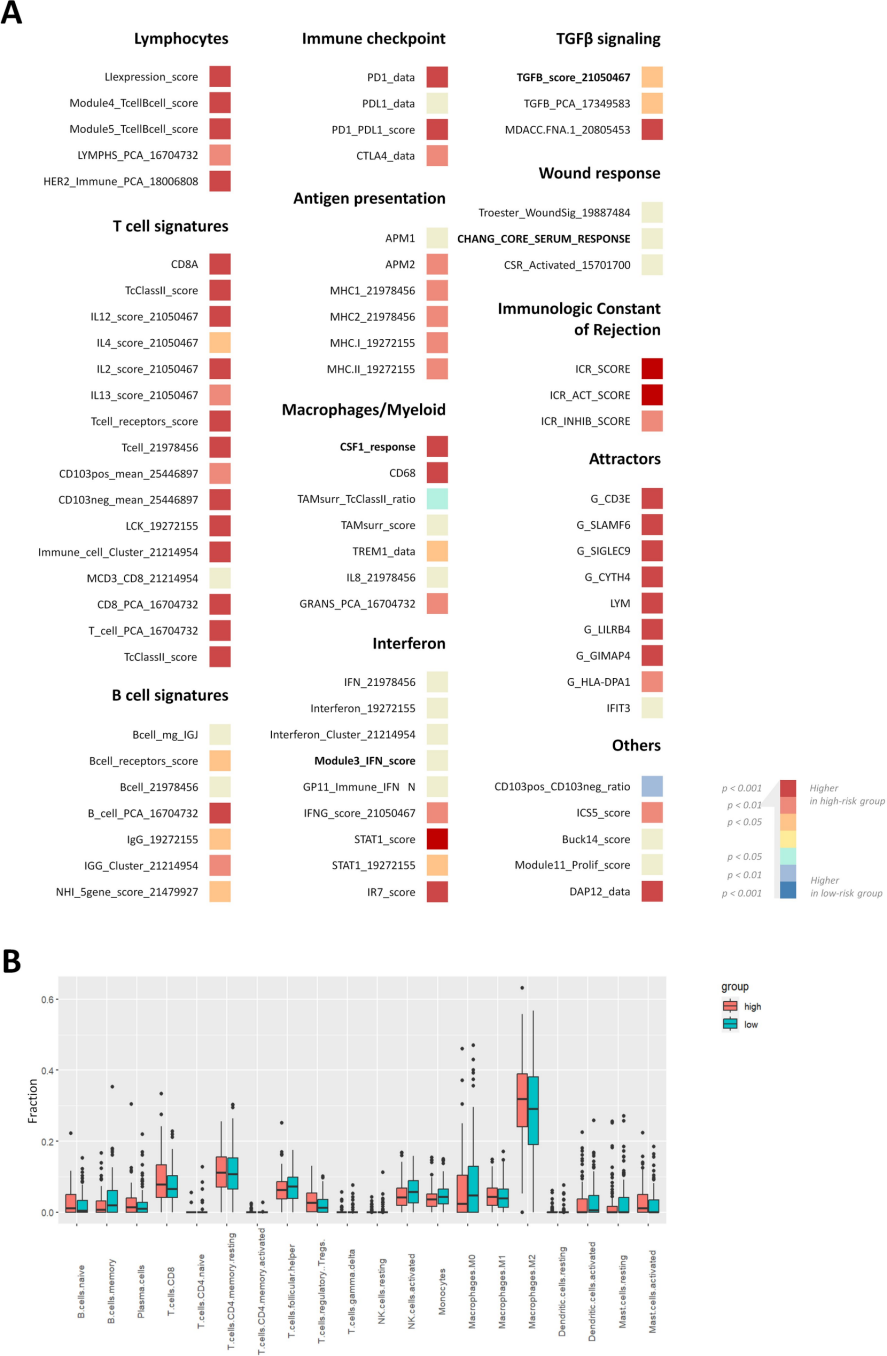
Deciphering the immune landscape between the risk groups. (**A**) Expression of 77 immunomodulatory signatures between the risk groups. Signatures exhibiting significantly higher expression in the high- and low-risk groups were indicated by red and blue colors, respectively, while signatures with no significant difference in expression were represented in yellow. (**B**) Box plot comparing the proportions of immune cells between the high- and low-risk groups

To comprehensively decipher the tumor immune microenvironments, 77 immunomodulatory signatures covering immune checkpoints, antigen presentation, lymphocytes, interferon pathway, wound response, and ECM dysregulation were included in our analysis (Fig. 7A). Out of the 77 signatures, 57 were significantly elevated in the high-risk group, while only 2 showed higher levels in the low-risk group. The 57 signatures were associated with T cell signatures, attractors, and the immunologic constant of rejection, suggesting a more active immunity. In comparison, the expression of B cell signatures and macrophages varied inconsistently between groups, and there were no significant between-group differences observed in most of the signatures related to interferon signaling and wound repair.

To further elucidate the differences in infiltrating immune cells between the risk groups, the CIBERSORT algorithm was used to estimate the proportions of 22 immune cells in OC samples. The proportions of several types of immune cells, including M2 macrophages (tumor-associated macrophages) and CD8 + T cells, were significantly higher in the high-risk group, while memory B cells were lower (Fig. 7B). Together these findings revealed distinct immune profiles associated with the risk groups.

### Assessment of immunotherapy responses using the risk score

**Fig. 8:**
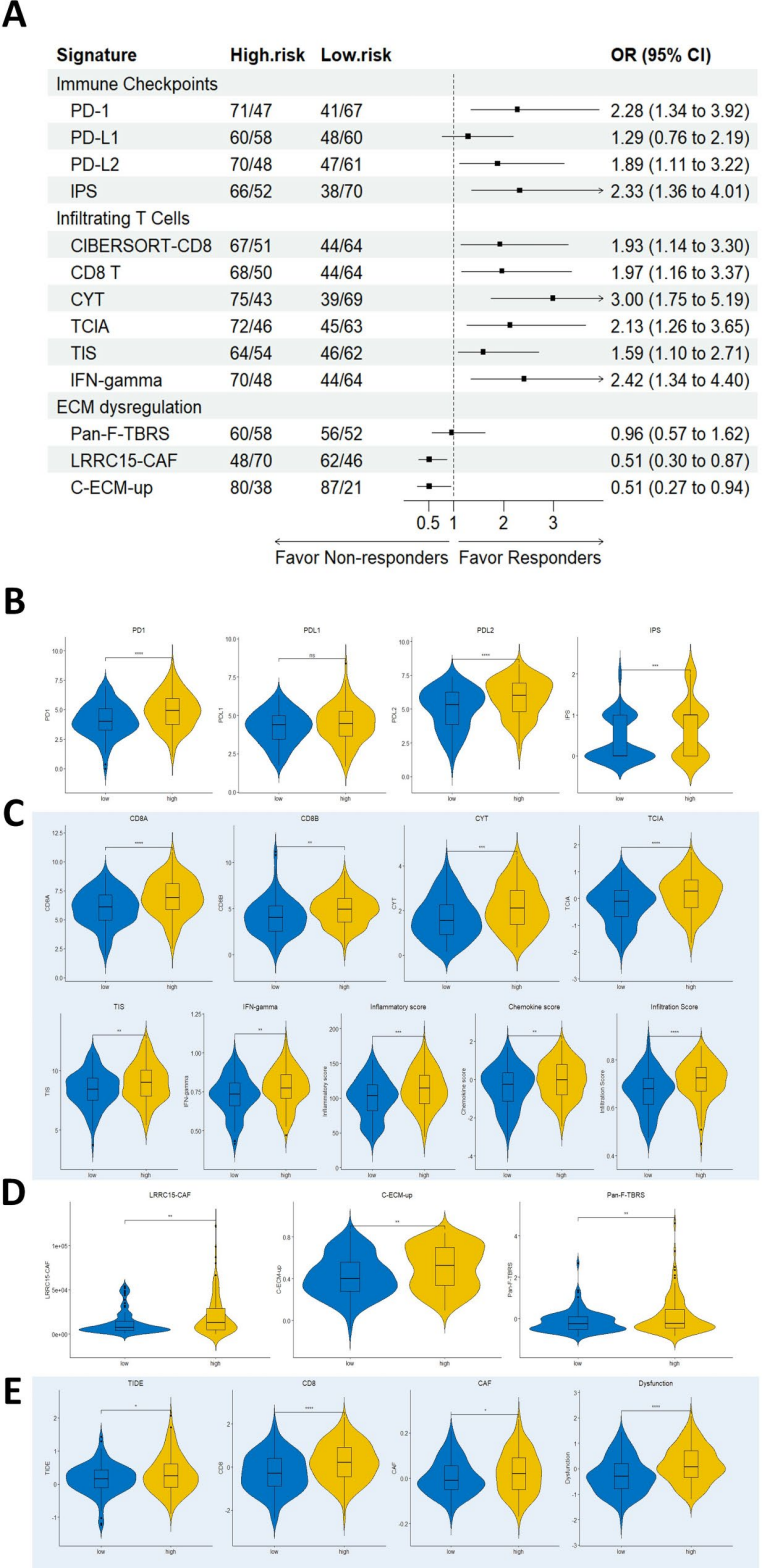
Assessing immunotherapy responses using the risk model. (**A**) The forest plot depicted the odds ratio (OR) and confidence interval of each signature for classifying the risk groups as either immunotherapy responders or non-responders. These published signatures are associated with (**B**) immune checkpoints, (**C**) infiltrating T cells, (**D**) ECM dysregulation, and obtained from (**E**) TIDE web application. The distributions of each signature between the risk groups were compared and visualized by box plots. *** p < 0.001, ** p < 0.01, * p < 0.05, *ns = non-significant*

Given the difference in infiltrating immune cells among the risk groups, we aimed to explore the potential of our risk score for immunotherapy response evaluation. To evaluate the ability of the risk score in identifying immunotherapy responders, we employed a variety of published signatures including those associated with immune checkpoints, infiltrating T cells, and ECM dysregulation. Patients were categorized as immunotherapy responders or non-responders based on each signature, and the odds ratio corresponding to each signature was summarized in Fig. 8A. A total of 266 OC patients with complete protein, RNA-seq data, and survival information were included. According to our findings, patients in the high-risk group were more likely to be classified as responders by immune checkpoint signatures, including Immunophenoscore (IPS) and gene expression levels of various immune checkpoints, such as PD-1 and PD-L2 (Fig. 8B).

In addition, the high-risk group demonstrated significantly higher levels of infiltrating T cell signatures, including IFN-gamma score, chemokine score, cytolytic activity score (CYT), and Tumor Inflammation Signature (TIS). These signatures were proposed to be positively associated with immunotherapy responses in various cancer types. Moreover, higher T cell-inflamed activity (TCIA), CIBERSORT-CD8, and CD8 T scores were observed in the high-risk group, indicating a T cell-inflamed microenvironment and a potentially greater proportion of immunotherapy responders in these patients (Fig. 8C). Additionally, we employed the ImmuCellAI algorithm to predict the susceptibility of patients to immunotherapy and found that the infiltration scores were significantly higher in the high-risk group, indicating a greater potential for immunotherapy (Fig. 8C). On the other hand, there were no significant differences in the odds ratio of predicted immunotherapy responders when OC patients were divided based on median TMB or HRD (Supplementary Figure S2). Collectively, compared to conventional indexes such as TMB, MSI, or HRD, our five-protein signature has greater potential to characterize a more inflamed immunophenotype in OC patients.

### TGF-β signaling mitigates the prognostic benefits of immune cell infiltration in the high-risk group

We extended our analysis to evaluate immunotherapy responses using signatures beyond CD8 T-cell immunity. To our surprise, we discovered that TGF-β-driven transcriptional signatures (signatures associated with ECM dysregulation), such as C-ECM up, Pan-F-TBRS, and LRRC15-CAF scores, were more likely to classify patients in the high-risk group as non-responders, as opposed to CD8 T-cell immunity signatures (Fig. 8D). Further investigation revealed a positive correlation between CD8 T-cell immunity and T-cell dysfunction signatures (r^2^ = 0.473, *p* < 0.001), and a higher TIDE score in the high-risk group (Fig. 8E). The high-risk group also demonstrated a higher abundance of cancer-associated fibroblasts (CAFs), which could potentially impair the cytotoxic T cells’ ability to eliminate cancer cells, thereby promoting tumor immune escape and contributing to the unfavorable prognosis observed in high-risk patients.

In summary, we have identified a set of proteins that are not only linked to the prognosis of OC patients but also correlated with both CD8 T-cell immunity and TGF-β signaling activation. The co-activation of TGF-β pathway in immunologically active tumors represents a mechanism of immune evasion, potentially leading to exclusion of CD8 T cells from the tumor tissue, ultimately diminishing the prognostic benefits of tumor immunogenicity [43]. Overcoming the ECM barrier is essential for improving the survival of the high-risk group identified by our five-protein signature. Combining novel therapeutics that target TGF-β signaling/cancer-associated fibroblasts with immunotherapy would be particularly beneficial for these OC patients [44].

## Discussion

The diverse nature and tumor microenvironments in OC pose significant challenges for personalized treatments, particularly in the realm of immunotherapy [44, 45]. While several clinical variables and gene expression signatures have shown promise as prognostic indicators, only a limited number of them have provided insights into both immunotherapy responses and prognoses [15, 17, 46, 47]. A previous study by Xu et al. failed to identify statistically significant survival differences when OC patients were categorized into immune-high and immune-low groups using 29 RNA-based immune gene sets. Similarly, our study also observed no significant differences in overall survival between the clusters defined by transcriptomic immune signatures (Fig. 2B). In this context, proteomics presents a complementary approach to genomics and transcriptomics, enabling the direct identification of effector molecules that influence the phenotype and cellular function of OC. Protein expression profiling also emerged as a valuable tool in understanding the interactions between the tumor and immune components in other cancers [20, 24]. However, the role of proteomic models in OC patients, particularly in assessing immunotherapy outcomes, remains largely unexplored.

Our study aimed to develop a protein-based signature to predict the prognosis and provide a comprehensive understanding of the immune landscapes in ovarian tumors. To achieve this, we began by clustering patients using immune-related gene sets, which allowed us to identify immune-related proteins of interest. Subsequently, through a series of feature selection including univariate Cox regression, LASSO, and multivariate Cox regression, we successfully developed a prognostic signature composed of 5 immune-related proteins and calculated the risk score for each patient. We divided patients into high-risk and low-risk groups by the median of the risk scores, with the low-risk group showing a longer overall survival than the high-risk group in both training and test sets. Our model demonstrated superior performance in assessing the prognosis compared to conventional clinicopathological characteristics such as age, grade, stage, MSI status, and HRD.

The five proteins in our signature are known to play crucial roles in OC. AR (Androgen receptor), a nuclear hormone receptor typically associated with male sexual development, has emerged as a significant player in OC [48]. AR signaling promotes tumor growth by stimulating genes involved in cell proliferation, survival, and angiogenesis. The prognostic implications of AR expression in OC are still being investigated [49–52]. FASN (Fatty acid synthase), an enzyme involved in de novo fatty acid synthesis, is frequently upregulated in OC. This altered lipid metabolism supports tumor growth by providing fatty acids for cell membrane formation and energy production. The dysregulation of FASN has been linked to a more aggressive tumor phenotype in in-vitro studies [53], but so far it has not been selected as a prognostic marker in transcriptomic signatures using real-world patient cohorts [54–57]. CDH2 (N-cadherin), a cell adhesion molecule, contributes to invasiveness and metastasis through epithelial-to-mesenchymal transition (EMT) [58]. CDH2 promotes EMT by reducing cell-cell adhesion and facilitating the detachment of tumor cells from the primary tumor, enabling their migration and invasion into surrounding tissues. MAPK14 (mitogen-activated protein kinase 14) is a protein kinase that plays a critical role in cellular responses to various stresses, including inflammation, oxidative stress, and DNA damage [59]. It can be tumor-suppressive or tumor-promoting depending on the context, and has been found to correlate with immune infiltration in colorectal cancer [60]. The aberrant MAPK signaling emerged as a potential target for OC [61]. PRKAA1 (AMP-activated protein kinase alpha 1) is an enzyme belonging to the AMPK family. Activation of PRKAA1 inhibits glycolysis, promotes mitochondrial function, induces cell cycle arrest, and reduces cell proliferation in ovarian tumor cells [62]. Collectively, these proteins represent the dysregulated pathways in ovarian tumors, and their reduced expression levels may serve as an indicator of lower tumor purity. This is supported by the positive correlation between the expression levels of AR, CDH2, PRKAA1 and tumor purity (Fig. 5B). Our study in TCGA OC patients demonstrated that low expression of these proteins is linked to an unfavorable prognosis. Our observation is consistent with Li et al.’s findings, where they identified five immune subtypes in OC based on immune-related gene expressions, and the subtype with the worst prognosis displayed low tumor purity and high fractions of leukocytes and stromal cells [63, 64]. The high-risk group in our study also showed significantly higher levels of CD8 T cell infiltration, as indicated by various immune-related signatures. This suggests that the expression of these proteins not only impacts tumor characteristics but also influences the immune microenvironment in OC. On the contrary, stratifying patients by TMB or HRD did not reveal significant differences in immune features, and stratifying patients by TMB or MSI did not show significant differences in survival (Supplementary Figure S2). Our findings reinforced the prognostic significance of non-cancerous cells within the tumor microenvironment, and successfully identified two subgroups with distinct prognoses and immune profiles.

To date, the association between infiltrating immune cells, PD-L1 expression, and the prognosis in OC patients varied among studies [65–67]. One possible explanation is the diversity in stromal components and TGF-β pathway, which has been implicated in promoting tumor immune escape and resistance to immunotherapy [68, 69]. For instance, in metastatic melanoma patients, the positive correlation between the abundance of cytotoxic T lymphocytes and improved survival is observed only when the expression level of TGF-β is low [42]. A CD8 + T cell/CAF ratio has recently been proposed as a more reliable indicator of treatment outcomes than PD-L1 [70]. In our study, the infiltrating immune cells did not confer prognostic benefits in TCGA OC patients. This observation may be attributed to the positive correlation between our risk score and both CD8 + T-cell immunity and TGF-β signatures. This aligns with previous research indicating that ovarian tumors with high TGF-β signaling tend to have worse outcomes [63]. We further examined the cell types known to restrict T cell function in tumors and found that the risk score positively correlates not only with the infiltration level of effector T cells in OC tumors but also with several immunosuppressive factors such as M2 macrophages and regulatory T cells. The higher degree of these immunosuppressive cells may hinder the effects of cytotoxic T cells and mitigate the prognostic benefits of infiltrating immune cells, resulting in poor outcomes in the high-risk group [43]. Therefore, despite being considered immunologically “hot” tumors based on CD8 T-cell signatures, the co-activation of the TGF-β pathway contributed to T-cell dysfunction and an unfavorable prognosis for the high-risk group [42]. These findings may provide insights into the lower overall response rates in OC patients treated with anti-PD-L1 therapy compared to other types of cancer, with the response rates of pembrolizumab and nivolumab being 11.5% and 15%, respectively [6, 71]. A combination strategy to overcome the immunosuppressive network is particularly necessary for OC, which can involve combining PD blockade with other agents such as PARP inhibitors or TGF-β inhibitors [44]. Gene expression profiles alone may not fully capture the comprehensive molecular mechanisms governing the regulation of the tumor immune microenvironments in ovarian tumors. Cancer Antigen 125 (CA125), the most widely-used protein biomarker for OC, has been shown to have clinical utility in assessing response to chemotherapy [72]. Moreover, a retrospective study by Boland et al. Memorial Sloan Kettering Cancer Center revealed that patients who benefit from immunotherapy had a smaller rise in CA-125 within 12 weeks, but the majority of responders experience an increase in CA-125 levels [73]. Caution should be warranted when using CA-125 levels to guide immunotherapy. On the other hand, our five-protein prognostic signature, which could be easily detected by immune-histochemical staining, may enable medical practitioners to assess individual prognoses and identify patients more likely to benefit from combinatorial therapies.

However, several limitations should be acknowledged. Firstly, the study is limited by a relatively small sample size and the lack of external validation. Secondly, while defining the risk groups based on the median risk score is straightforward and commonly used, it may not be the most effective for identifying optimal cutoff. For generalizability and simplicity, we chose the median as the cutoff because the alternative method proposed by Contal et al. yielded similar stratification results [74]. Lastly, there is a lack of CA-125 information in the TCGA cohort, highlighting the need to include the well-established biomarkers in future research. Additional studies are warranted to validate and refine this signature and explore its underlying molecular mechanisms as well as clinical applications in guiding immunotherapy strategies for OC patients.

## Conclusions

In summary, we developed a protein-based signature for predicting the prognoses of OC patients. The five-protein signature effectively identified distinct subgroups linked to prognoses and immune profiles. Our risk model defined a specific group of patients characterized by increased immune cell infiltration but poor outcomes. Overcoming the immunosuppressive microenvironments is crucial for these patients to fully leverage the potential of immunotherapy.

## Electronic supplementary material

Below is the link to the electronic supplementary material.

Supplementary Material 1

Supplementary Material 2

## Data Availability

All data used in this study can be obtained from public sources. The RPPA protein expression profiles and RNA-sequencing data of OC patients were downloaded from The Cancer Proteome Atlas (TCPA) database (https://tcpaportal.org/tcpa/) and the pan-cancer atlas of The Cancer Genome Atlas (TCGA) program (https://gdc.cancer.gov/about-data/publications/pancanatlas). The clinical information and mutation profiles, including TMB, MSI, and HRD, were retrieved from the TCGA pan-cancer immune landscape (https://gdc.cancer.gov/about-data/publications/panimmune).
