# Correction: Novel protein-based prognostic signature linked to immunotherapeutic efficiency in ovarian cancer

**DOI:** 10.1186/s13048-025-01605-6

**Published:** 2025-01-28

**Authors:** Shuo-Fu Chen, Liang-Yun Wang, Yi-Sian Lin, Cho-Yi Chen

**Affiliations:** 1 Department of Heavy Particles & Radiation Oncology, Taipei Veterans General Hospital, Taipei, 112 Taiwan; 2 Institute of Biomedical Informatics, National Yang Ming Chiao Tung University, Taipei, 112 Taiwan; 3 Program in Genetics and Genomics, Baylor College of Medicine, Houston, TX 77030 USA; 4 Brain Research Center, National Yang Ming Chiao Tung University, Taipei, 112 Taiwan

**Correction:** ***J Ovarian Res*** **17, 190 (2024)**

10.1186/s13048-024-01518-w

Following publication of the original article [1], the authors reported that the funding section was not included in the published article.

**Funding**

This work was supported by the Ministry of Science and Technology and the National Science and Technology Council of Taiwan (MOST111-2634-FA49-014, MOST111-2636-E-A49-006, MOST 111-2221-E-A49-130-MY3, NSTC112-2320-B016-008) and the Ministry of Health and Welfare (MOHW112-TDU-B-222-124013).

The original article [1] has been corrected.
