# Supplementary material for: Novel protein-based prognostic signature linked to immunotherapeutic efficiency in ovarian cancer
Source: J Ovarian Res. 2024 Sep 28;17:190. doi: 10.1186/s13048-024-01518-w (PMC11437962; doi:10.1186/s13048-024-01518-w)
Supplement: Supplementary file 2 — Supplementary Material 2 [file 13048_2024_1518_MOESM2_ESM.pdf]

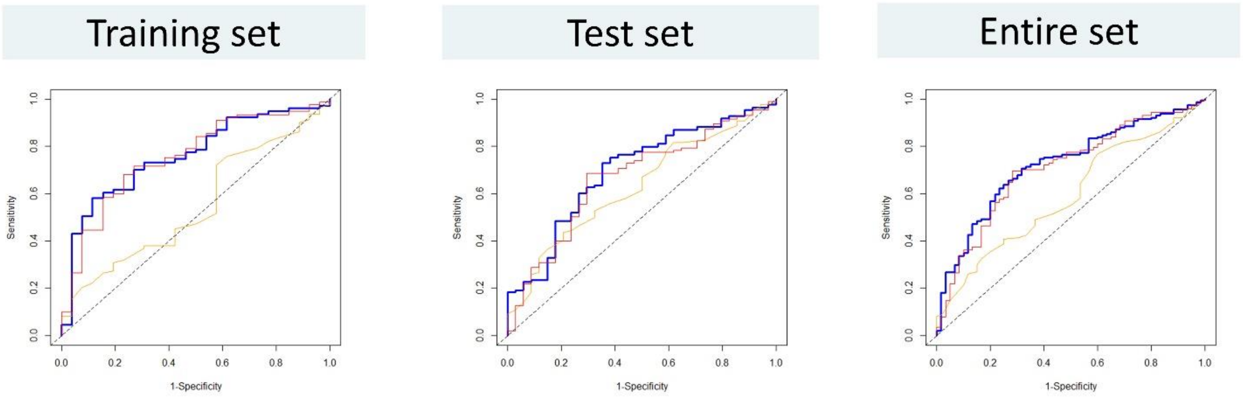

	Training set	Test set	Entire set
Risk score	75.5	66.7	70.9
Age	54.8	63.0	59.2
Risk score + Age	76.1	69.7	72.5

Supplementary Figure S1. Prognostic performance of the model combining age and the proteomic signature.

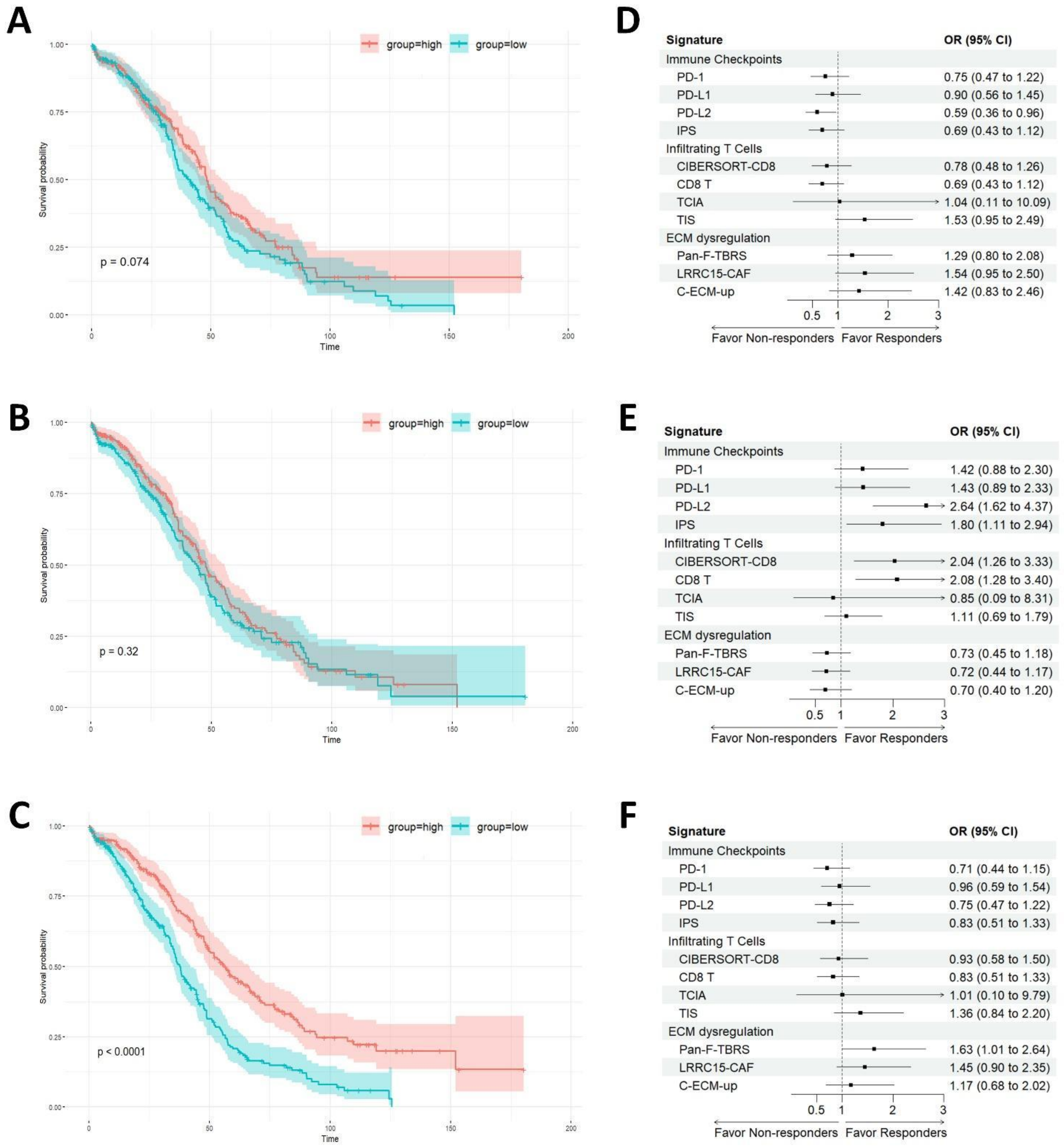

Supplementary Figure S2. The Kaplan–Meier analysis of overall survival when patients were stratified by the median (A) TMB (B) MSI, and (C) HRD. The odds ratio (OR) of predicted immunotherapy responders when patients were divided based on median (D) TMB, (E) MSI, and (F) HRD.
